# Therapeutic Effect of Gypenosides on Antioxidant Stress Injury in Orbital Fibroblasts of Graves' Orbitopathy

**DOI:** 10.1155/2022/4432584

**Published:** 2022-09-15

**Authors:** Chao Ma, Haoyu Li, Wei Liu, Shuwen Lu, Xian Li, Jinyuan Chen, Kaijun Li, Wenzhan Wang

**Affiliations:** ^1^The First Affiliated Hospital of Zhengzhou University, China; ^2^The First Affiliated Hospital of Guangxi Medical University, China; ^3^The First Affiliated Hospital of Henan University of Chinese Medicine, China; ^4^University of Manchester, UK; ^5^The First Affiliated Hospital of Fujian Medical University, China

## Abstract

**Purpose:**

To examine the impact of gypenosides (Gyps) on oxidative stress damage of orbital fibroblasts (OFs) from Graves' ophthalmopathy (GO) patients.

**Methods:**

The relationship between Gyps and GO oxidative stress was understood by bioinformatics analysis. Orbital connective tissues of GO and non-GO patients were obtained for primary OF culture. The proliferation level of OFs was measured by Cell Counting Kit-8 method, and the appropriate intervention concentration of Gyps and H_2_O_2_ was obtained. The expression of apoptosis-related protein mRNA was analyzed by RT-qPCR technique. ROS and SOD test suites were employed to detect the oxidative stress level in OFs. Flow cytometry apoptosis detection, TUNEL detection, and lactate dehydrogenase detection were used to analyze the level of apoptosis. Western blotting detection was utilized to examine the regulatory pathway of oxidative stress, apoptosis, and autophagy-related proteins. The changes of cell morphology, autophagosome, and autophagy lysosome were observed by transmission electron microscope.

**Results:**

The suitable intervention concentration of Gyps is 100 *μ*g/mL, and the suitable intervention concentration of high concentration H_2_O_2_ is 350 *μ*M. In comparison with the blank control group, the H_2_O_2_ intervention group enhanced the expression of apoptosis-related mRNA, the expression of ROS and SOD, the apoptosis rate, the expression of autophagy activation-related protein and Nrf2/ERK/HO-1 protein, and the number of autophagosomes and autophagy lysosomes. Compared with H_2_O_2_ intervention group, the expression of apoptosis-related mRNA decreased, ROS expression decreased, SOD expression increased, apoptosis rate decreased, autophagy activation-related protein expression decreased, Nrf2/ERK/HO-1 protein expression increased, and the quantity of autophagosomes and autophagy lysosomes decreased in H_2_O_2_ + Gyps intervention group.

**Conclusion:**

Gyps can decrease the oxidative stress level of OFs generated by H_2_O_2_, reduce cell autophagy, and reduce apoptosis. Gyps may regulate the oxidative stress response of OFs in GO patients via the Nrf2/ERK/HO-1 signaling pathway.

## 1. Introduction

Graves' orbitopathy (GO), also recognized as thyroid associated orbital disease or thyroid ophthalmopathy, is the most prevalent extrathyroid symptom of Graves' disease (GD) [1]. The annual incidence is 20-50/1000,000, but the incidence is as high as 50% in GD patients [2, 3]. With the progress of the disease, there may be exophthalmos, eyelid retraction, limited eye movement, and even loss of vision [4] . Therefore, GO is a serious and potentially irreversible disease that affects the autoimmune process of orbital tissue, which can reduce patients' quality of life even if the condition is mild. Although the pathogenesis and pathological process are not fully understood, oxidative stress has a significant role in the pathogenesis of the disease. Several investigations have found that antioxidants play an antioxidant role in orbital fibroblasts (OFs) at the cellular level, leading to decreased cell proliferation and hyaluronic acid (HA) secretion, which are the two main mechanisms of GO pathogenesis [5]. The use of antioxidants such as selenium can prevent some orbital microenvironmental changes caused by oxidative stress, especially by reducing the release of some cytokines associated with the pathogenesis of GO [6, 7]. In addition, antioxidants also have the effect of antiproliferation and can also reduce the cytotoxicity caused by oxidative stress, thus reducing the possible release of orbital autoantigen, thus triggering or aggravating the autoimmune response to orbital tissue [8]. Furthermore, in OFs of the patients with GO, the increase of reactive oxygen species (ROS) or the decrease of scavenging free radicals by antioxidant enzymes will lead to oxidative damage of cell membrane, lipid peroxidation, and DNA oxidative damage, leading to local inflammatory response and functional impairment [9]. Moreover, it was also found that the index of oxidative stress increased, and the levels of lipid hydrogen peroxide, superoxide dismutase (SOD), glutathione reductase, and glutathione peroxidase in orbital fibrous connective tissue increased significantly, whereas the production of antioxidant glutathione decreased. Glutathione level and ophthalmopathy index had a substantial negative correlation [10]. Therefore, oxidative stress is crucial in the GO pathogenesis and orbital lesions.

OFs can proliferate and differentiate into myofibroblasts and adipocytes and also generate extracellular matrix, which causes tissue edema and fibrosis [11–13]. Their interactions with mononuclear cells, as well as the production of various chemical inducers and cytokines, result in permanent orbital pathological changes [14]. Therefore, they are regarded as essential effector cells that play an important part in the pathogenesis of GO. Research has revealed that oxidative stress injury is one of the important pathogenesis of GO [15, 16]. An increase in superoxide free radicals can enhance the development of OFs and glycosaminoglycan synthesis [17]. Moreover, it can lead to oxidative damage of cell membrane, lipid peroxidation, and oxidative DNA damage, leading to local inflammatory reaction and cell function change or loss [9]. Hydrogen peroxide (H_2_O_2_), a naturally reactive oxygen species formed by human cells throughout physiological and pathological processes, has been employed as an oxidant in the research of oxidative stress-related disorders [18]. To examine the protective effects of drugs on oxidative stress injury, H_2_O_2_ was used to establish oxidative stress model.

Gypenosides (Gyps) are saponin extract from Gynostemma pentaphyllum. It has the effects of antihypertension, antiaging, antihyperlipidemia, antihyperglycemia, and anti-inflammation [19–22]. It is a traditional Chinese herbal medication used to treat chronic inflammation, hyperlipidemia, and cardiovascular disease [23]. And in a variety of diseases, modern experimental methods have been used to prove that it has the effect of antioxidation. Gyps can protect the heart of rats with myocardial infarction by maintaining myocardial SOD activity and decreasing myocardial malondialdehyde (MDA) level [24]. It is reported that Gyps may also effectively protect organs from ischemia-reperfusion injury through its antioxidant activity [25, 26]. In addition, Gyp preintervention limits the size of myocardial infarction in rats with myocardial ischemia-reperfusion, and this protection is followed by a decrease in oxidative stress and the protection of mitochondrial function [27]. However, prior studies have not identified the role of Gyps in regulating oxidative stress in GO.

ERK-activated Nrf2/HO-1 signal pathway is a crucial protective mechanism against oxidative stress and an important antioxidant stress pathway [28, 29]. In diabetic retinopathy, curcumin activates ERK/Nrf2/HO-1 signal pathway, protecting retinal pigment epithelial cells from injury induced by high glucose level [30]. The Nrf2/HO-1 signal activation involves the activation of the ERK pathway, which improves cell antioxidant defense and reduces inhibits stress to protect C2C12 myoblasts from H_2_O_2_-induced oxidative cytotoxicity [31]. By activating this route, several antioxidants, including flavonoids, polyphenols, and carotenoids, can perform an antioxidant function [32–34]. And recent studies have shown that polydatin can decrease ROS production by stimulating the Nrf2-ARE pathway, thereby reducing the oxidative stress-related symptoms in GO [35]. However, it is not clear whether Gyps can also activate this pathway.

In view of the above, the purpose of this research was to investigate the impact of Gyps on OFs cultured in vitro. We analyzed whether Gyps can reduce the injury of OFs in patients with GO caused by oxidative stress. We further discussed the relevant mechanism.

## 2. Materials and Methods

### 2.1. Data Acquisition and Preprocessing

From the GEO database (https://www.ncbi.nlm). The data sets of GO and non-GO in GSE58331 were collected (http://www.ncbi.nlm.nih.gov/geo/query/acc.cgi?acc=GSE58331; visit on June 15, 2019). Use the GEOquery package in GEO2R to read the data, and the limma package in GEO2R to normalization and difference analysis; in this step, we select genes with *P* < 0.05 and ∣log2FC | ≥1. The array consists of 22 GO samples and 20 non-GO samples. Anterior orbit and lacrimal gland tissues were collected for RNA extraction and analysis with Affymetrix microarrays. Biopsy samples were obtained from subjects with inflammatory diseases such as NSOI, sarcoidosis, GPA, and TED as wells as from normal controls. Gyp constituents were obtained from the Traditional Chinese Medicine System Pharmacology Database and Analysis Platform (TCMSP, http://lsp.nwu.edu.cn/tcmsp.php), a distinct systematic pharmacological platform built for Chinese herbal medicine. The action targets of Gyps were predicted by the method of pharmacophore interaction under three-dimensional model through PharmMapper website (http://www.lilab-ecust.cn/pharmmapper/).

### 2.2. Bioinformatics Analysis

For the differential genes obtained by GO and the predicted target of Gyps, Venny 2.1 (http://bioinfogp.cnb.csic.es/tools/venny/index.html) was utilized to obtain the intersection target. Database for Annotation, Visualization, and Integrated Discovery (DAVID v6.8 and https://david.ncifcrf.gov/) was then utilized to analyze the GO, KEGG, and Reactome pathways of the intersection targets. In order to further understand the complex relationship of interaction and close relationship between intersection targets, a protein-protein interaction network (PPI) was developed based on the information of STRING v11.0 (https://string-db.org/). We choose 0.10 as the threshold of the common target. At the same time, node 1, node 2, and combination score of string database are transferred into the Cytoscape 3.7.1 software, and the visualization of PPI network is constructed by setting node size and color to map values and score values.

### 2.3. Reagents and Chemicals

Gyps were obtained from the Xi'an Jiatian Biotech Co., Ltd. (China, purity 98%). 3% H_2_O_2_ was obtained from the Millipore (California, USA). Dulbecco's modified Eagle's medium (DMEM), fetal bovine serum (FBS), penicillin, and streptomycin were obtained from Invitrogen (California, USA). The Cell Counting Kit-8 (CCK-8) assay was obtained from Beyotime Biotechnology (Shanghai, China). PCR kit and its primer synthesis were obtained from Takara (Dalian, China). The ROS, SOD, MDA, and lactic dehydrogenase (LDH) kits were obtained from Solarbio (Beijing, China). Primary antibodies targeting anti-B cell leukemia 2 (Bcl-2), anti-beclin 1 (BECN1), anti-caspase-3, anti-microtubule-associated protein 1 light chain 3*α* (LC 3), anti-sequestosome 1 (p62), and anti-*β*-actin antibodies were all obtained from Cell Signaling Technology (Massachusetts, USA). Polyvinylidene difluoride (PVDF) membranes were obtained from Solarbio Life Sciences (Beijing, China). BSA was purchased from Dalian Meilun Biotechnology (Dalian, China).

### 2.4. Subject Recruitment

Orbital connective tissue was acquired from 5 GO patients (female 3, male 2, age 48 -- 61 years old) who underwent orbital decompression with an average clinical activity score (CAS) of 1.2. For the control group, 4 patients (female 2, male 2, age 21 -- 50 years old) underwent eyeball enucleation or upper eyelid blepharoplasty. All the patients with GO achieved stable thyroid function during the operation, and their CAS was less than 3 during the operation [36]. Subjects who had orbital radiotherapy or systemic steroids during the preceding three months were excluded. Subjects with strabismus or cavernous hemangioma were included in the control group, but subjects with other orbital inflammatory diseases were excluded.  Table 1 provides detailed clinical information. The study followed the principles of the Helsinki Declaration, each subject signed a written informed consent, and the Hospital Ethics Review Committee has approved this study.

### 2.5. Cell Culture

The OFs were isolated and extracted from the tissue obtained during the operation by the method of tissue mass primary culture, and the OFs were cultured as mentioned previously. In short, the tissue was chopped and placed directly in DMEM: F12 (1 : 1) medium containing 20% fetal bovine serum, penicillin (100 U/mL), and streptomycin (0.1 mg/mL). The culture medium was placed in 5% CO_2_ humidifier at 37°C. The primary cells were then digested and subcultured in a 25 cm flask containing DMEM, antibiotics, and 10% fetal bovine serum with trypsin/ethylenediamine tetraacetic acid (EDTA). Some of the cells were preserved in liquid nitrogen while cultured, and only the cells between the third generation and the eighth generation were utilized for follow-up research.

### 2.6. Cell Vitality

OFs were inoculated in 96-well plate at a density of 6000/well, cultured in 5% CO_2_ incubator at 37°C for 24 h, and then treated with different concentrations of H_2_O_2_ (H_2_O_2_: 50, 150, 250, 350, and 450 *μ*M) for 24 h, or treated with Gyps in different concentrations (Gyps: 25, 50, 100, 250, and 500 *μ*g/mL) for 48 h. The cells were then incubated with CCK-8 reagent (10 *μ*L/well) for 3.5 h, and the cell viability was detected. The absorbance at 450 nm was determined using a full-wavelength enzyme labeling instrument to evaluate cell proliferation. All of the processes are conducted in compliance with the manufacturer's instructions.

### 2.7. Real-Time Quantitative PCR (RT-qPCR)

Based on the manufacturer's instructions, TRIzol was used to extract total RNA, which was subsequently reverse transcribed into cDNA. Real-time quantitative PCR (RT-qPCR) amplification was conducted with SYBR PreMix Ex TaqII (Takara Biotechnology, Dalian, China) to quantitatively evaluate the level of gene transcription in cell samples. All PCR reactions were performed in triplicate. The primer sequence is as follows: BAX: CGAACTGGACAGTAACATGGAG (forward) and CAGTTTGCTGGCAAAGTAGAAA (reverse); caspase-3: CCAAAGATCATACATGGAAGCG (forward) and CTGAATGTTTCCCTGAGGTTTG (reverse); and GAPDH: GACAGTCAGCCGCATCTTCT (forward) and GCGCCCAATACGACCAAATC (reverse). All samples are adjusted to their corresponding GAPDHs; results were expressed by the relative multiple change of the threshold period (Ct) value compared to the control group, utilizing the 2 − *ΔΔ*Ct method.

### 2.8. Biochemical Analysis Related to Oxidative Stress

ROS includes hydrogen peroxide and its downstream products, which are involved in several physiological and pathological processes of cells. The fluorescent dye 2B7-dichlorofluorescein diacetate (DCFH-DA) was utilized as the substrate to observe the fluorescent substance formed by the compound and ROS for quantitative analysis. In short, the cells incubated 25 min in the dark at 37°C with 10 *μ*M of DCFH-DA. Then, wash it three times with serum-free medium. The fluorescence value was detected by full-wavelength enzyme labeling instrument at excitation wavelength of 488 nm and emission wavelength of 525 nm. For image shooting, fluorescent cells are displayed using a fluorescence microscope (Olympus, Tokyo, Japan).

SOD exists widely in cultured cells and catalyzes the disproportionation of superoxide anions, which is not only the superoxide anion scavenging enzyme but also the main H_2_O_2_ producing enzyme. First, collect 106 cells into the centrifuge tube, add 200 *μ*L extract, ultrasonic fragmentation (power 20% or 200 W, ultrasound 3 s, interval 10 s, and repeat 30 times), then 8000*g* 4°C centrifugation for 10 minutes, get the supernatant. After fully mixing with the working solution on a 96-well plate, after 30 min in a water bath at 37°C, the absorbance of each tube was measured at 560 nm by full-wavelength enzyme labeling instrument.

By detecting the level of MDA, we detect the level of lipid peroxidation caused by the activity of oxygen free radicals on the unsaturated fatty acids of lipids. 106 cells were transferred into a centrifuge tube, and the supernatant was removed after centrifugation; 250 *μ*L extract was added, and the ultrasound was used to break the cells (power 20%, ultrasound 3 s, interval 10 s, and repeated 30 times); 8000*g* centrifuged at 4°C for 10 minutes, and the supernatant was obtained. After detecting the concentration of protein, add the prepared working liquid and place 60 minutes in a 100°C water bath pot (cover tightly to prevent moisture loss) and cool in an ice bath. Then, centrifuge 10 minutes with 10000*g* at room temperature. The samples were packed into 96-well plates, and the absorbance of each sample was detected by 450 nm, 532 nm, and 600 nm.

### 2.9. Cell Apoptosis and Cycle Assay

In order to observe changes in apoptosis, we carried out Annexin V-FITC/PI double staining experiment. The cells were washed with PBS twice and then resuspended and mixed with 500 *μ*L buffer, the final concentration was 1 × 10^6^ cells/mL, and then, 5 *μ*L Annexin V-APC and 10 *μ*L 7-AAD were added to each tube to incubate 5 minutes at room temperature. The (BD Biosciences, San Jose, CA, USA) of stained cells was examined by FACS flow cytometry.

Apoptosis of OFs was detected by TUNEL method. 4% paraformaldehyde was fixed at room temperature for 30 minutes, PBS with 0.3% Triton X Mel 100 was permeated at room temperature for 5 minutes and then incubated in a TUNEL reaction mixture at 37°C for 60 minutes, and the nucleus was stained with DAPI for 5 minutes, then sealed with an antifluorescence attenuation tablet, and then observed under a fluorescence microscope.

LDH can be released from the cell after injury, and cell mortality is further assessed by measuring leakage into the surrounding culture medium, as described in previous studies. The supernatant of the culture medium was collected, the reagent was prepared and added strictly according to the manufacturer's instructions, the incubated mixed liquid was packed into a 96-well plate, and the absorbance was observed at 450 nm by full-wavelength enzyme labeling instrument.

To observe changes in the cell cycle, we collect 5 × 10^5^ cells and centrifuge to discard the supernatant. Wash once with PBS and centrifuge to discard the supernatant. Add 1 mL DNA staining solution and 10 *μ*L permeabilization solution, vortex, and mix for 5 seconds. Incubate at room temperature in the dark for 30 minutes. Select the lowest sample loading speed and test on the flow cytometer.

### 2.10. Western Blotting Assay

After washing with PBS, the cells were scraped into an EP tube containing ice cell lysis buffer and phenylmethanesulfonyl fluoride (PMSF), and the cells were cracked on the ice for 10 s by ultrasonic cell crusher and then placed in the ice box for 1 hour, reversing and mixing once every 10 minutes. Then, centrifuge at 4°C and 12000*g* for 20 minutes; the supernatant was proportionally added to the protein buffer and boiled. 10% SDS-PAGE gel was prepared for electrophoresis, the isolated protein was transferred into the polyvinylidene fluoride membrane, BSA was closed for 1 hour, and the primary antibody was incubated overnight at 4°C before being treated for 1 hour with fluorescent secondary antibody. Fluorescence gel imaging analysis instrument was used for scanning and analysis.

### 2.11. Transmission Electron Microscope

Prepare 3 × 10^6^ cells for intervention, digest with trypsin containing EDTA, centrifuge at 1500 rpm at 4°C, resuspend and transfer to a 1.5 mL EP tube, centrifuge again to remove the supernatant according to the above conditions, and add 2.5% E cells fixed with dialdehyde phosphate buffer and 1% osmium tetroxide. Wash with 0.1 mol/L phosphate buffer 3 times, 15 minutes each time, then fix with 1% osmium acid for 2 hours, wash with 0.1 mol/L phosphate buffer 3 times, 15 minutes each time, and then use ethanol-acetone grade dehydration: use 50% ethanol, 70% ethanol, 90% ethanol, 1 : 1 mixture (90% ethanol: 90% acetone), 90% acetone, and 3 times 100% acetone (15 minutes each time). Then, use acetone and embedding agent at a ratio of 1 : 1 for more than 2 hours; then, use a ratio of 1 : 3 for more than 3 hours, and finally, fully soak the pure embedding agent overnight, and then, use epoxy resin 618 for embedding; polymerization was performed at 40°C for 15 hours, 48°C for 12 hours, and 60°C for 24 hours. Finally, semithin sections were produced and double-stained using uranyl acetate and lead citrate. Transmission electron microscopy was used to observe the ultrastructure of cells as well as the typical structures and quantities of autophagosomes and autophagolysosomes after staining.

### 2.12. Statistical Analysis

The data were computed by the GraphPad Prism (Windows v8.2.0) software (GraphPad Software, San Diego, USA). In order to compare the data between the GO group and the control group, two-way ANOVA test was used, and multiple comparisons were made. One-way ANOVA was used to compare the results within the GO group, and multiple comparisons were done between the groups. All the experiments were carried out using at least three different samples of cells, each in duplicate. The *P* value of statistical analysis is reported in Results and the illustration, and the *P* value of multiple comparisons is shown as an asterisk (∗ as *P* < 0.05 and ∗∗ as *P* < 0.01). For *P* values less than 0.05, differences were deemed to be statistically significant.

## 3. Results

### 3.1. Data and Bioinformatics Analysis

**Figure 1:**
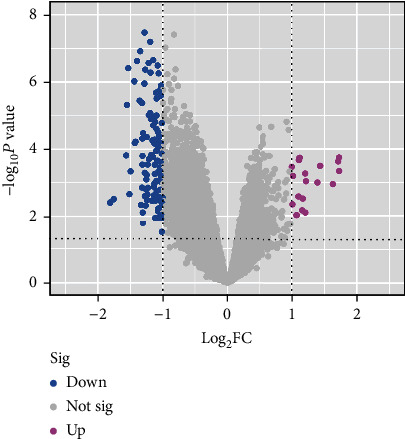
The volcanic map of differentially expressed genes of GSE58331; red dots and green dots represent upregulated and downregulated genes, respectively. *P* < 0.05, ∣log2FC | ≥1.

**Figure 2:**
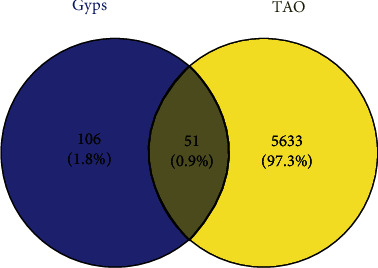
Venn diagram obtained the common target of thyroid-associated ophthalmopathy and gypenoside saponins by intersection.

**Figure 3:**
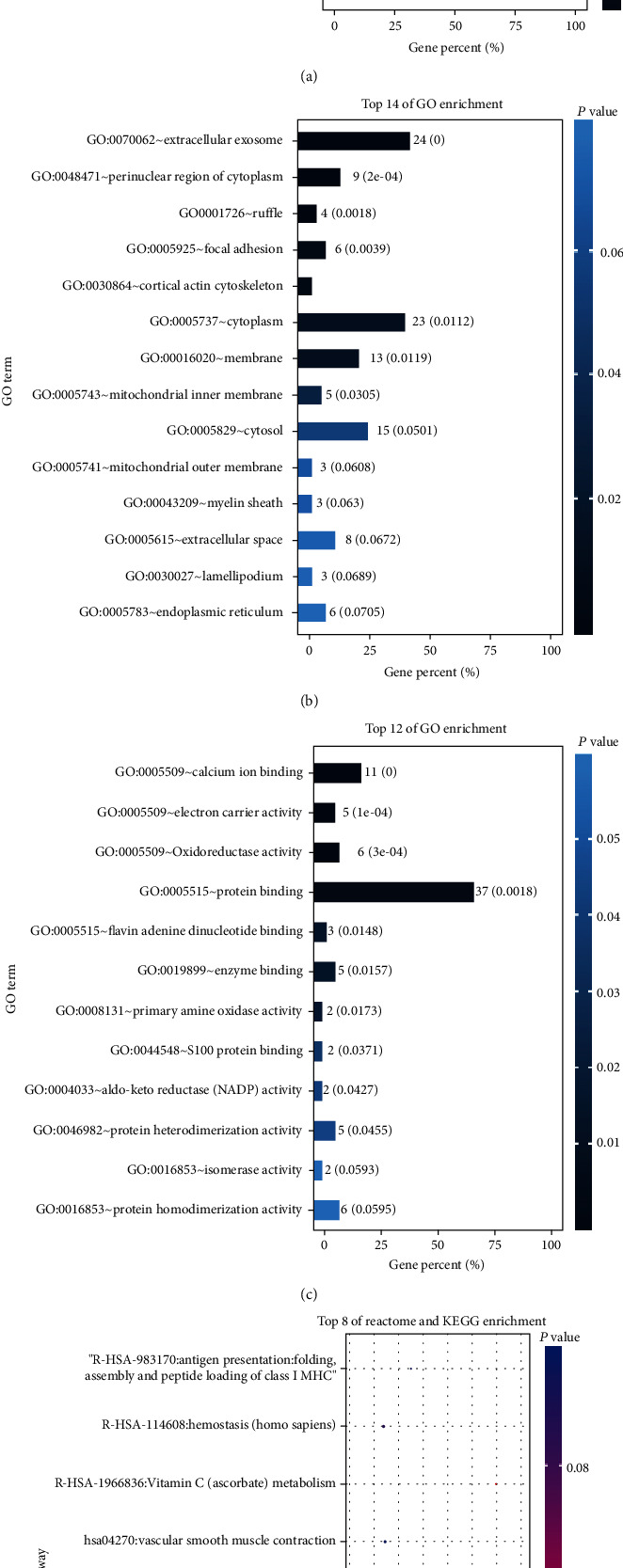
Gene Ontology (GO) and pathway enrichment analysis: (a) biological process; (b) cell composition analysis; (c) molecular function; (d) Reactome and Kyoto Encyclopedia of Genes and Genomes (KEGG) pathway enrichment analyses.

**Figure 4:**
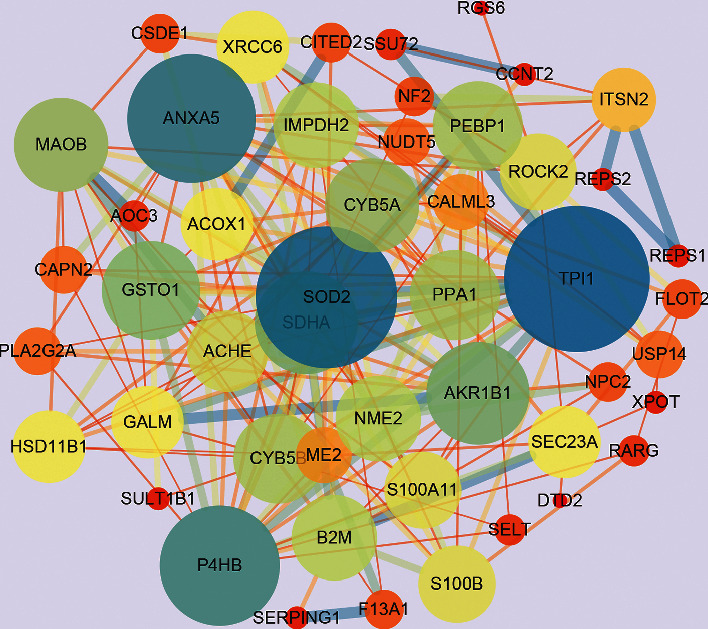
Construction of protein-protein interaction network for common target protein expression of GO and gypenosides. The 46 nodes represent 46 proteins, and the 197 edges represent the interactions between 197 pairs of proteins. The node size and color represent the degree, and the edge size and color represent the comprehensive score.

5684 differential genes (Figure 1) of tissue microarray of GO patients and non-GO patients were obtained by using GSE58331 dataset in GEO database. Through the TCMSP website, we identified 104 Gyps and then used the PharmMapper website to predict 157 gene targets. The coexpressed gene (Figure 2) was obtained by intersection of the two. In the subsequent GO analysis of the common targets, we found the gene targets related to the biological process and pathogenesis of Gyps and GO, mainly include GO:0055114 ~ oxidation-reduction process, GO:0043066 ~ negative regulation of apoptotic process, GO:0001666 ~ response to hypoxia, GO:0016491 ~ oxidoreductase activity, and GO:0008131 ~ primary amine oxidase activity. As for the pathway analysis, it was mainly related to metabolic pathway (hsa01100) and vitamin C antioxidant pathway (R-HSA-196836) (Figure 3). Additionally, in the following protein-protein interaction network analysis, it was found that SOD2 is the most important position in the network and is closely related to other proteins (Figure 4).

### 3.2. Detection of Cytotoxicity by CCK-8

**Figure 5:**
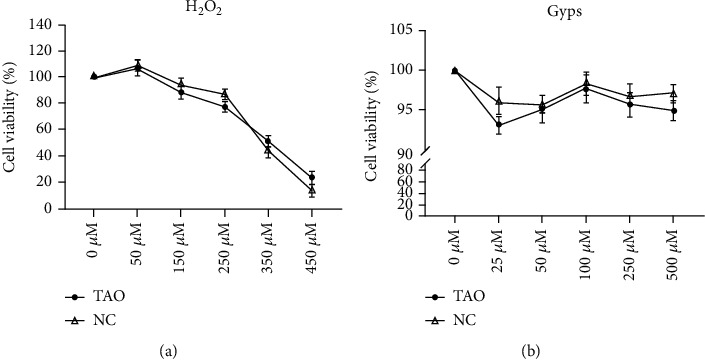
Effects of H_2_O_2_ and gypenosides on the viability of orbital fibroblasts in patients with GO and non-GO. (a) Treating OFs in GO and non-GO patients with 350 *μ*M H_2_O_2_ for 24 hours can achieve 50% viability of cells, which is an appropriate concentration to establish apoptosis model. (b) Treating OFs of GO and non-GO patients with 100 *μ*g/mL Gyps for 48 hours can reach the maximum cell viability and not exceed the intervention concentration of the control group.

The in vitro efficacy of Gyps and H_2_O_2_ on OFs in GO patients and non-GO patients was determined by CCK-8 colorimetric method. First of all, OFs were treated with various doses of H_2_O_2_ for about 24 hours, and the effect of H_2_O_2_ on apoptosis was observed (Figure 5(a)). Except that the cell activity increased under the stimulation of 50 *μ*M H_2_O_2_, the activity of OFs decreased under the stimulation of other concentrations of H_2_O_2_ and decreased to about 50% at 350 *μ*M. Then, OF was treated with various doses of Gyps for 48 h (Figure 5(b)). All concentrations of Gyps inhibited the proliferation of OFs, and cell proliferation was much greater in the 100 *μ*g/mL group than in the 25 and 500 *μ*g/mL groups (univariate analysis of variance). Therefore, in this study, 350 *μ*M H_2_O_2_ was selected to induce oxidative stress and apoptosis of OFs, and 100 *μ*g/mL Gyps was used as a protective agent to study its role in antioxidation and antiapoptosis in OFs of GO patients.

### 3.3. Gyps Inhibit Apoptosis of OFs in GO and Non-GO Patients Induced by H_2_O_2_

**Figure 6:**
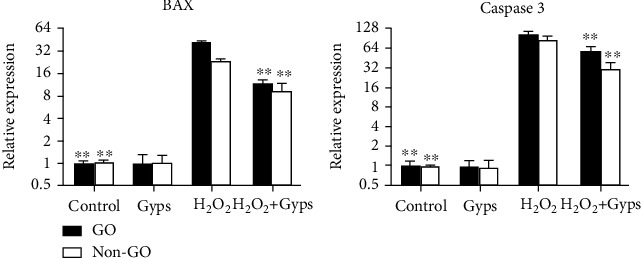
Effect of gypenosides on the production of apoptosis-related protein and autophagy-related protein mRNA in orbital fibroblasts of patients with GO. Intervention was carried out according to different groups. The extracted RNA was reverse transcribed by real-time fluorescence quantitative PCR. Preintervention of gypenosides could significantly inhibit the mRNA expression of caspase-3 and BAX in orbital fibroblasts of GO and non-GO patients. Three samples from each of the three GO and non-GO donors were taken for the experiment. All detections are repeated 3 times, with 3 replicate wells each time (^∗^ as *P* < 0.05 and ^∗∗^ as *P* < 0.01; *P* < 0.05 means the difference is statistically significant, and *P* value is analyzed by two-way ANOVA).

In order to observe whether the in vitro intervention of Gyps on OFs in GO patients and non-GO patients is the same, real-time fluorescence quantitative PCR was utilized to observe the transcription of caspsase-3 and BAX in GO and non-GO OFs stimulated by H_2_O_2_ for 24 hours after preintervention with Gyps for 24 hours (Figure 6).

### 3.4. Gyps Can Regulate the Level of Oxidative Stress in OFs

**Figure 7:**
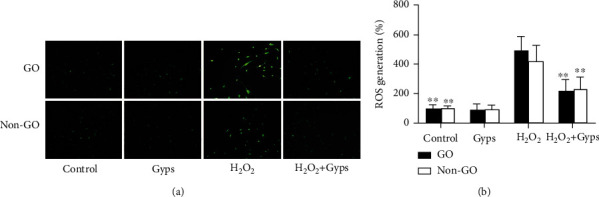
Effect of gypenosides on the activity of superoxide dismutase induced by H_2_O_2_. Gypenoside preintervention can downregulate the level of reactive oxygen species in orbital fibroblasts of GO patients under H_2_O_2_ stimulation. After intervention in accordance with the requirements of the group. The orbital fibroblasts of GO patients in each treatment group were localized in situ by ROS fluorescence intensity, and then, (a) was photographed by fluorescence microscope. And the fluorescence values of each group of ROS were detected after the excitation light and emission light were adjusted by full-wavelength enzyme labeling instrument. The result is expressed as a percentage of unprocessed control values. Three samples from each of the three GO and non-GO donors were taken for the experiment. All detections are repeated 3 times, with 3 replicate wells each time (^∗^ as *P* < 0.05 and ^∗∗^ as *P* < 0.01; *P* < 0.05 means the difference is statistically significant, and *P* value is analyzed by two-way ANOVA).

**Figure 8:**
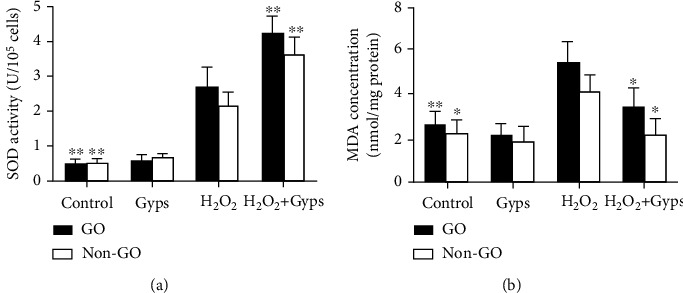
The effect of Gyps on SOD and MDA induced by H_2_O_2_. Gypenoside preintervention can regulate the expression of SOD and MDA in orbital fibroblasts of GO patients under H_2_O_2_ stimulation. After intervention in accordance with the requirements of the group. SOD and MDA kits were used to detect (a) in each group with full-wavelength enzyme labeling instrument. SOD showed that the activity of H_2_O_2_ + Gyps increased significantly, while MDA detection showed that H_2_O_2_ + Gyps could inhibit its production. Three samples from each of the three GO and non-GO donors were taken for the experiment. All detections are repeated 3 times, with 3 replicate wells each time (^∗^ as *P* < 0.05 and ^∗∗^ as *P* < 0.01; *P* < 0.05 means the difference is statistically significant, and *P* value is analyzed by two-way ANOVA).

We studied whether Gyps act as an antioxidant in OFs from GO patients and non-GO patients. When the cells were treated with Gyps and H_2_O_2_ at the above concentrations, ROS, SOD, and MDA were detected. The ROS ratio and MDA expression of the Gyp group were lower compared to the control group, and the SOD activity of the Gyp group was greater than that of the control group, but the difference was insignificant. However, the amount of ROS produced by the H_2_O_2_ + Gyp group was significantly lower compared to the H_2_O_2_ treatment group (*P* < 0.01, Figure 7). At the same time, the activity of SOD in the H_2_O_2_ + Gyp group was higher compared to that in the H_2_O_2_ group (*P* < 0.01, Figure 8(a)). In MDA detection, the expression level of the H_2_O_2_ + Gyp group was lower compared to the H_2_O_2_ group (*P* < 0.05, Figure 8(b)).

### 3.5. Gyps Have a Protective Effect on Apoptosis Induced by H_2_O_2_

**Figure 9:**
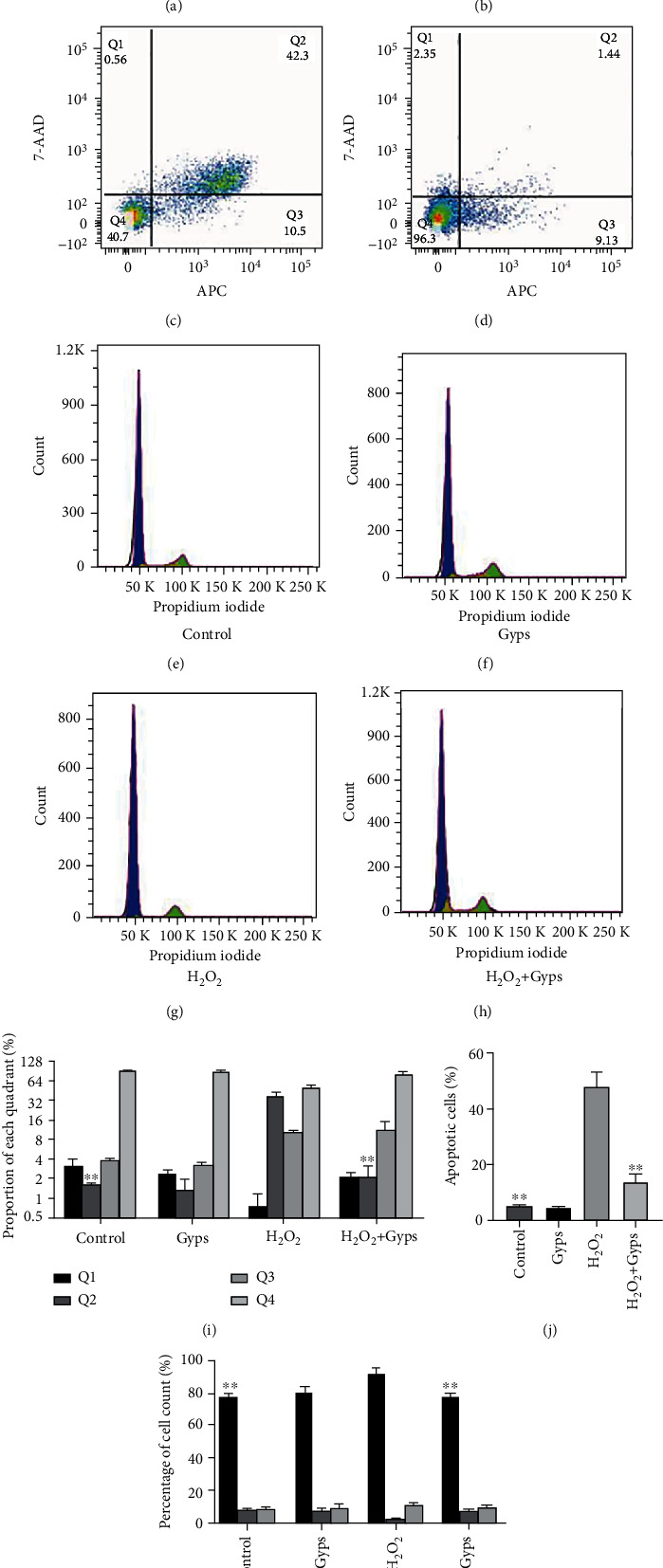
The effect of gypenosides on apoptosis and cycle induced by H_2_O_2_. Flow cytometry was used to analyze the effects of gypenosides on H_2_O_2_-induced apoptosis and cell cycle. (a–d) Flow cytometry detection. (e–h) Flow cytometry. (i) Results of cell ratio in 4 regions. (j) Results of cell apoptosis rate in each group. (k) Results of cell cycle in each group. Three samples from each of the three GO donors were taken for the experiment. All detections are repeated 3 times, with 3 replicate wells each time (^∗^ as *P* < 0.05 and ^∗∗^ as *P* < 0.01; *P* < 0.05 means the difference is statistically significant, and *P* value is analyzed by one-way ANOVA).

**Figure 10:**
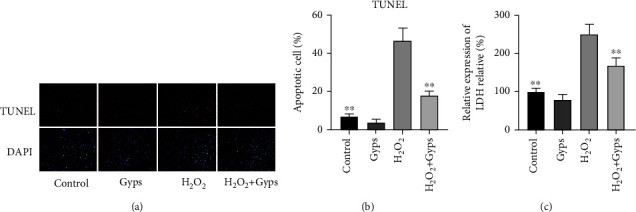
Effect of Gyps on late apoptosis induced by H_2_O_2_. (a) Fluorescence photograph. (b) Results of apoptosis rate of each group. (c) The absorbance of each group was quantitatively measured by full-wavelength enzyme labeling instrument using LDH detection kit. The results showed that the apoptosis of H_2_O_2_ + Gyps was significantly decreased. Three samples from each of the three GO donors were taken for the experiment. All detections are repeated 3 times, with 3 replicate wells each time (^∗^ as *P* < 0.05 and ^∗∗^ as *P* < 0.01; *P* < 0.05 means the difference is statistically significant, and *P* value is analyzed by one-way ANOVA).

In order to study the antiapoptotic effect of Gyps under oxidative stress injury, we detected the apoptosis and cycle of flow cytometry, the apoptosis of TUNEL, and the relative expression of LDH secreted by cells. Flow cytometry showed that Gyps could significantly reduce the late apoptotic cells but had little effect on the early apoptotic cells. The overall data revealed that the apoptosis rate in the H_2_O_2_ + Gyp group was lower compared to the H_2_O_2_ group (*P* < 0.01, Figure 9). Cell cycle analysis showed that the proportion of G0/G1 phase in cell cycle in the H_2_O_2_ + Gyp group was lower compared to that in the H_2_O_2_ group (*P* < 0.01, Figure 9). The results of apoptosis detection of TUNEL cells showed that the apoptosis rate of the H_2_O_2_ + Gyp group was lower compared to the H_2_O_2_ group (*P* < 0.01, Figures 10(a) and 10(b)). The results of membrane integrity and cytotoxicity test with LDH reported that the relative expression of the H_2_O_2_ + Gyp group was significantly lower compared to the H_2_O_2_ group (Figure 10(c)).

### 3.6. Effects of Gyps on Nrf2/ERK/HO-1 Signaling Pathway, Apoptosis, and Autophagy-Related Proteins

**Figure 11:**
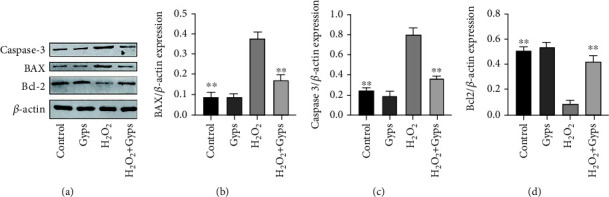
Gypenosides can protect the apoptosis induced by H_2_O_2_. The results showed that compared with the blank control group, the expression of caspase-3 and BAX increased, and the expression of Bcl-2 decreased after H_2_O_2_ treatment. Compared with H_2_O_2_ intervention group, the expression of caspase-3 and BAX decreased, while Bcl-2 increased in preintervention of gypenosides was further increased. These bands are quantified by densitometry and then normalized to the level of *β*-actin in the same sample. All proteins were expressed by the ratio of band density to *β*-actin band density. Three samples from each of the three GO donors were taken for the experiment. All detections are repeated 3 times, with 3 replicate wells each time (^∗^ as *P* < 0.05 and ^∗∗^ as *P* < 0.01; *P* < 0.05 means the difference is statistically significant, and *P* value is analyzed by one-way ANOVA).

**Figure 12:**
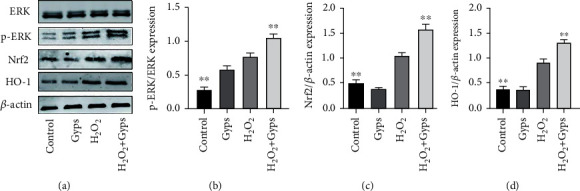
Gypenoside has a protective effect on H_2_O_2_-induced oxidative stress through the ERK/Nrf2/HO-1 signaling pathway. The results showed that compared with the blank control group, the expression of p-ERK, Nrf2, and HO-1 increased after H_2_O_2_ intervention. Compared with the H_2_O_2_ group, the expression of p-ERK, Nrf2, and HO-1 in preintervention of gypenosides was further increased. (b) Each represents the value obtained by dividing the band density of the phosphorylated transcription factor by the same total band density of the transcription factor. On the other hand, (c) and (d) are expressed by the ratio of band density to *β*-actin band density. Three samples from each of the three GO donors were taken for the experiment. All detections are repeated 3 times, with 3 replicate wells each time (^∗^ as *P* < 0.05 and ^∗∗^ as *P* < 0.01; *P* < 0.05 means the difference is statistically significant, and *P* value is analyzed by one-way ANOVA).

**Figure 13:**
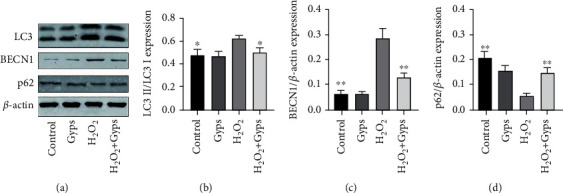
Gypenosides can reduce autophagy induced by H_2_O_2_. The results showed that compared with the blank control group, the expression of LC3II and BECN1 increased, and the expression of p62 decreased after H_2_O_2_ treatment. Compared with the H_2_O_2_ intervention group, the expression of LC3II and BECN1 decreased, while p62 increased in preintervention of gypenosides was further increased. These bands are quantified by densitometry and then normalized to the level of *β*-actin in the same sample. (b) The value obtained by dividing the band density of the transcription factor of LC3 II by the band density of LC3 I. On the other hand, (c) and (d) are expressed by the ratio of band density to *β*-actin band density. Three samples from each of the three GO donors were taken for the experiment. All detections are repeated 3 times, with 3 replicate wells each time (^∗^ as *P* < 0.05 and ^∗∗^ as *P* < 0.01; *P* < 0.05 means the difference is statistically significant, and *P* value is analyzed by one-way ANOVA).

In order to investigate the impact and mechanism of Gyps on antioxidant stress and antiapoptosis under oxidative stress, we observed the influences of Gyps on the expression of apoptotic proteins, Nrf2/ERK1/2/HO-1 signal pathway, and autophagy-related proteins. Gyps at 100 *μ*g/mL could regulate the expression of apoptotic proteins in OFs of GO patients stimulated by H_2_O_2_. Caspase-3 and BAX expression rose in the H_2_O_2_ group compared to the control group, but Bcl-2 expression dropped. In the H_2_O_2_ + Gyp group, caspase-3 and BAX expression reduced while Bcl-2 expression rose when compared to the H_2_O_2_ group (Figure 11). The observation of the Nrf2/ERK/HO-1 signaling pathway revealed that Nrf2, p-ERK, and HO-1 expressions increased in the H_2_O_2_ group compared to the control group, while the expressions of Nrf2, p-ERK, and HO-1 in the H_2_O_2_ + Gyp group were considerably greater than those in the H_2_O_2_ group (Figure 12). In terms of autophagy-related proteins, the expressions of LC3 and BECN1 in the H_2_O_2_ group increased, and the expression of p62 decreased compared to the control group. Furthermore, in the H_2_O_2_ + Gyp group, LC3 and BECN1 expressions dropped while p62 expression rose when compared to the H_2_O_2_ group (Figure 13). Every experiment was carried out in three GO cells from distinct patient samples, with each cell sample examined in duplicate.

### 3.7. Gyps Can Reduce the Number of Autophagosomes and Autophagolysosomes

**Figure 14:**
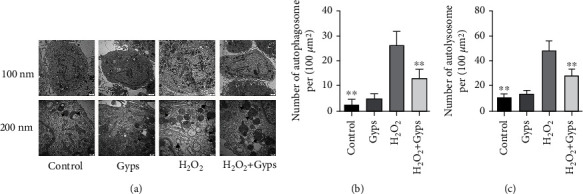
Transmission electron microscopy was used to detect autophagy expression in orbital fibroblasts. (a) Electron micrograph. Arrows indicate autophagosomes, and triangles indicate autophagolysosomes. (b) H_2_O_2_ stimulation can increase the number of autophagosomes, and the number of autophagosomes in the H_2_O_2_ + Gyp intervention group is lower than that in the H_2_O_2_ group. (c) H_2_O_2_ stimulation can increase the number of autophagolysosomes, and the number of autophagolysosomes in the H_2_O_2_ + Gyp intervention group is lower than that in the H_2_O_2_ group. Three samples from each of the three GO donors were taken for the experiment. All detections are repeated 3 times, with 3 replicate wells each time (^∗^ as *P* < 0.05 and ^∗∗^ as *P* < 0.01; *P* < 0.05 means the difference is statistically significant, and *P* value is analyzed by one-way ANOVA).

Typical autophagosomes and autolysosomes can be detected by transmission electron microscopy. In the control group OFs, the organelles and nuclei were clearly organized, while in the H_2_O_2_ group, nuclear fragmentation was seen, and the number of autophagosomes and autolysosomes increased significantly. In comparison with the H_2_O_2_ group, the number of autophagosomes and autophagolysosomes in the H_2_O_2_ + Gyp group was significantly reduced (Figure 14).

## 4. Discussion

Bioinformatics analysis revealed that SOD2 was the target protein with the greatest enrichment score in this research, while GO analysis and pathway analysis revealed that oxidative stress-related pathways were strongly associated to Gyps and GO. These results indicate that Gyps may affect GO by regulating the oxidative stress process. In addition, we observed the effects of H_2_O_2_ on OFs oxidative stress, apoptosis, and autophagy in GO patients. Under the stimulation of 350 *μ*M H_2_O_2_, Gyps can reduce the generation of ROS and the cell autophagy by regulating the ERK/Nrf2/HO-1 signaling pathway and then play an anti-OF apoptosis role. Therefore, Gyps can inhibit the damage of OFs induced by oxidative stress.

SOD2 is a ribocoding enzyme, which is a member of the SOD family, and finally transferred to the mitochondrial matrix of cells after transcription and translation. In the mitochondrial matrix, Mn is added to the catalytic site of SOD2, giving the enzyme the ability to conduct dismutase activity [37, 38]. Among various ROS defense mechanisms, SOD is a key antioxidant enzyme and is involved in a variety of human diseases. SOD can resist O2- produced in the body, produce molecular oxygen or H_2_O_2_, and then be degraded to H_2_O. As we know, oxidative stress is an important factor in the pathogenesis and progression of GO. Therefore, as a part of antioxidative stress, SOD may play an antioxidative stress role in the orbital tissue of GO patients under the regulation of Gyps.

GO is a kind of autoimmune disease that involves both endogenous and exogenous factors [39]. Activated T lymphocytes invading the orbit are believed to trigger a series of processes after detecting the common antigens of the thyroid and orbit, culminating in an increase in the production of cytokines, extracellular matrix, and ROS [40]. The stimulation of exogenous H_2_O_2_ further aggravated the imbalance of preexisting redox state in GO fibroblasts. The increase of DNA damage caused by oxidative stress in GO patients is associated to the clinical evolution of the disease, especially inflammatory activity [41]. Previous research has suggested that H_2_O_2_ has a bidirectional impact on OFs. When the dose of H_2_O_2_ is greater than 5 *μ*M, it shows cytotoxicity, which reflects the direct cytotoxicity of H_2_O_2_ to cells [5]. In addition, in vitro and in vivo studies of OFs emphasize that ROS can promote orbital changes [42]. Therefore, to examine the regulatory effect of Gyps on oxidative stress of OFs, this study used 350 *μ*M H_2_O_2_ to stimulate OFs to induce apoptosis and observe the protective effect of Gyps. The results showed that after preintervention with Gyps, the expression of ROS and MDA of OFs stimulated by H_2_O_2_ was significantly reduced, and the antioxidative stress index SOD was significantly increased. Flow cytometry and TUNEL tests showed that apoptotic cells were significantly reduced and LDH apoptosis-related detection indicators significantly decreased, resulting in significantly reduced levels of proinflammatory cytokines and extracellular matrix. Therefore, Gyps can inhibit the oxidative stress response of OFs in GO patients and protect the damage caused by oxidative stress to OFs. It is unclear whether Gyp has the ability to activate the ERK/Nrf2/HO-1 pathway. The findings of this research indicate that Gyps can upregulate p-ERK expression, causing Nrf2 expression to increase and increase HO-1 expression, suggesting that it may activate the ERK/Nrf2/HO-1 signaling pathway. It laid the foundation for the follow-up mechanism research.

Autophagy is an important process of lysosomal degradation that leads to aging and damaged cellular components. It can produce energy and nutrients required for intracellular homeostasis and function [43]. Kirkland et al. [44] found that a small amount of ROS accumulation in cells can clear the damaged organelles by increasing autophagy, but a high concentration or long-term accumulation of ROS can stimulate excessive activation of autophagy and directly cause cell death. In summary, autophagy serves two functions. A certain degree of autophagy helps protect cells, while too much autophagy might harm cells or tissues. Autophagy and apoptosis also have a strong connection. Some studies have shown that taurine can reduce retinal cell apoptosis by inhibiting autophagy [45]. Animal experiments also proved that H_2_O_2_ treatment of cells can increase the autophagy of MRI skin fibroblasts and lead to apoptosis by inhibiting the PI3K/AKT pathway [46]. Although there are few reports on the relationship between GO and autophagy, autophagy has been shown to cause liver fibrosis, which is similar to the similar pathological features of GO [47]. In addition, the autophagosome structure observed under transmission electron microscopy is the standard method to prove the autophagy of OFs [48]. Therefore, we conducted transmission electron microscopy on the OFs of each group. Morphologically, a large number of free membrane structures appear in the cytoplasm when cells undergo various types of autophagy. These model structures continue to expand and surround the substances to be degraded to form autophagic bodies. The results of electron microscopy coincide with the trend of Western blot results. Therefore, in this study, we found that Gyps may reduce the oxidative stress damage caused by H_2_O_2_ by regulating autophagy-related pathways, and it has a protective impact on OFS in GO patients.

In the results of the cell cycle, we found that after using H_2_O_2_ to induce OFs, the G0/G1 phase of the cell was blocked to inhibit cell proliferation, and after the preintervention with Gyps, the G0/G1 phase block decreased, and the cell restored its proliferative ability. These are consistent with the trend of apoptosis results we have obtained. And some studies have shown that Quinitan can exert a stable antitumor effect by promoting G0/G1 phase blockade, which can affect liver cancer cell apoptosis [49] . Moreover, the antifibrotic drug pirfenidone may inhibit pancreatic cancer cell proliferation by promoting G0/G1 phase arrest [50]. There is growing evidence that ROS is a key regulator of cell division and differentiation [51]. The connection between ROS and cell cycle arrest has previously been identified [52]. A few researches show that 4-amino-2-trifluoromethylphenyl retinate can significantly stimulate leukemia cells to release ROS. ROS scavenger N-acetylcysteine and ferrotitanium reagent can inhibit 4-amino-2-trifluoromethylphenyl retinate-induced leukemia cell differentiation and G0/G1 phase arrest. These results indicate that ROS contribute in the differentiation induced by 4-amino-2-trifluoromethylphenyl retinate and G0/G1 phase arrest [53]. Therefore, the cell cycle results indicate that Gyps have the ability in regulating the cell cycle, alleviating the G0/G1 phase blockade, and reducing the apoptosis of OFs in protecting ROS-induced apoptosis.

Gyps have antioxidant and antiapoptotic effects in many diseases. In the study of Parkinson's disease, Gyps are able to decrease the expression level of LDH, Bax, cytochrome c, caspase-3-9, and PPAR and increase the expression level of Bcl-2. In order to protect PC12 cells from loss of activity induced by 1-methyl-4-phenylpyridine ion and inhibit apoptosis [54]. Gyps can prevent retinal ganglion cell apoptosis and enhance final visual acuity in patients with optic neuritis through antioxidation and immunomodulation, indicating that it may have neuroprotective and immunomodulatory properties [55]. Recently, GPM extracts Gyp LVII, Gyp J1, Gyp J2, and Gyp J3 had significant protective effects on oxidative stress induced by H_2_O_2_ in human neuroblastoma SH-SY5Y cells [56]. Gyp L and Gyp Li may suppress lung cancer cell A549 growth and metastasis by causing apoptosis, blocking the cell cycle during the G0/G1 phase, and reducing cell migration [57]. These studies support the antioxidant and antiapoptotic effects of Gyps.

Since traditional Chinese medicine has a systemic immunomodulatory impact, it is critical to investigate the anti-inflammatory mechanism of traditional Chinese medicine [58]. In the research of osteoporosis, Gyps can suppress osteoclast development induced by the NF-B, Akt, and MAPK signaling pathways [59]. Additionally, by blocking the NF-*κ*B signaling pathway, Gyps can suppress the inflammatory response of osteoarthritis chondrocytes generated by IL-1*β* [60]. In terms of antioxidant stress, Gyps can directly reduce the content of intracellular ROS and protect cells from apoptosis induced by H_2_O_2_, thus enhancing the resistance of retinal ganglion cells to oxidative damage [61]. Gyps can also significantly improve the antioxidant capacity of the body, reduce lipid peroxidation products and DNA oxidative damage, decrease inflammatory astrocytes activation, and have the potential to be employed as a treatment for dementia caused by chronic cerebral hypoperfusion [62]. Low density lipoprotein-treated retinal pigment epithelial cells promote the occurrence of age-related macular degeneration by activating NF-*κ*B signal pathway and increasing the production of ROS and proinflammatory cytokines. Gyps can treat individuals with early age-related macular degeneration by promoting cholesterol clearance in retinal pigment epithelial cells and inhibiting inflammation and oxidative stress [63]. Moreover, Gyps can prevent hypoxia-induced nerve injury by activating extracellular regulated protein kinase, protein kinase B, and cyclic adenosine monophosphate response element binding protein signal pathway [64]. Therefore, Gyps serve as a plant extract and has the potential to become an excellent medication for the treatment of GO patients.

## 5. Conclusion

**Figure 15:**
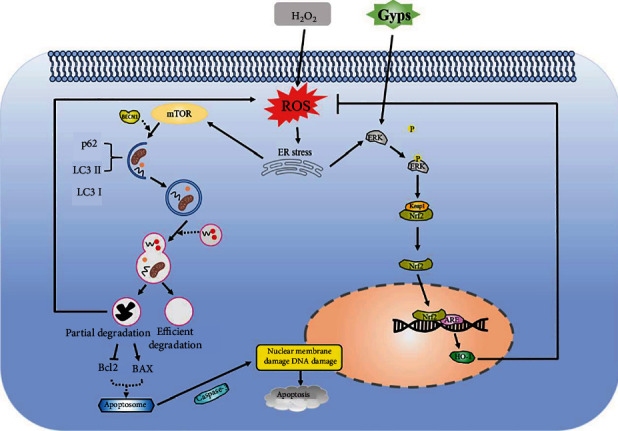
Schematic diagram of changes in orbital fibroblasts under the intervention of oxidative stress and gypenosides. Under the condition of oxidative stress, the increase of endogenous ROS leads to the occurrence of endoplasmic reticulum stress. On the one hand, endoplasmic reticulum stress induces the occurrence of autophagy. If the oxidative stress damage is serious, autophagy cannot fully digest the damaged organelles. It will lead to increased ROS production and promote the start of apoptosis-related processes leading to apoptosis. On the other hand, endoplasmic reticulum stress can promote ERK phosphorylation, allowing Nrf2 to interact with its inhibitor under oxidative stress, causing Nrf2 to accumulate in the cytoplasm, and Nrf2 translocated to the nucleus and combined with antioxidant response elements. Gypenosides as an antioxidant can enhance the role of endogenous Nrf2 pathway activator. Under oxidative stress, gypenosides can promote ERK phosphorylation and accelerate the dissociation of Nrf2 from Keap1, resulting in more Nrf2 translocation to the nucleus. NRF2 binds through antioxidant response elements and regulates the expression of its downstream target gene (HO-1), to prevent oxidative stress and damage.

In conclusion, Gyps have antioxidant, antiapoptotic, anti-inflammatory, and antifibrosis effects on OFs from GO and non-GO patients stimulated by H_2_O_2_ in vitro. Gyps reduced ROS and increased SOD, to restore redox to equilibrium in vivo. By regulating autophagy (Figure 15), it can inhibit OF apoptosis and the production of proinflammatory cytokines and fibrosis-related extracellular matrix. More study is necessary to prove Gyps' function in the clinical setting. The optimal dose and more effective method of transporting Gyps to OFs in vivo also need to be further studied.

## Figures and Tables

**Table 1:** Disease information and clinical characteristics of patients taking orbital tissue samples.

Characteristics	GO		Non-GO	
Gender	3 males, 3 females		2 males, 2 females	
Age	Patient 1	61	Patient 1	35
	Patient 2	48	Patient 2	21
	Patient 3	52	Patient 3	50
	Patient 4	49	Patient 4	33
	Patient 5	57	—	—
	Patient 6	63	—	—
Smoker	Patient 1	No	Patient 1	No
	Patient 2	No	Patient 2	No
	Patient 3	No	Patient 3	Yes
	Patient 4	Yes	Patient 4	No
	Patient 5	No	—	—
	Patient 6	No	—	—
BMI	Patient 1	22.8	Patient 1	22.6
	Patient 2	22.9	Patient 2	23.6
	Patient 3	25.8	Patient 3	23.9
	Patient 4	20.3	Patient 4	21.3
	Patient 5	19.5	—	—
	Patient 6	22.8	—	—
Duration of GO (years)	Patient 1	0.5	Patient 1	—
	Patient 2	0.5	Patient 2	—
	Patient 3	1	Patient 3	—
	Patient 4	1	Patient 4	—
	Patient 5	20	—	—
	Patient 6	0.6	—	—
CAS	Patient 1	0/7	Patient 1	—
	Patient 2	1/7	Patient 2	—
	Patient 3	1/7	Patient 3	—
	Patient 4	3/7	Patient 4	—
	Patient 5	1/7	—	
	Patient 6	1/7	—	
Proptosis R/L (mm)	Patient 1	21/20	Patient 1	—
	Patient 2	20/22	Patient 2	—
	Patient 3	19/21	Patient 3	—
	Patient 4	20/18	Patient 4	—
	Patient 5	23/23	—	—
	Patient 6	21/19	—	—
Surgical treatment	Patient 1	Decompression	Patient 1	Eye evisceration
	Patient 2	Decompression	Patient 2	Eye evisceration
	Patient 3	Decompression	Patient 3	Eye evisceration
	Patient 4	Decompression	Patient 4	Upper lid blepharoplasty
	Patient 5	Decompression	—	—
	Patient 6	Decompression	—	—

GO: Graves' orbitopathy; BMI: body mass index; CAS: clinical activity score; R: right eye; L: left eye.

## Data Availability

The original contributions presented in the study are included in the article; further inquiries can be directed to the corresponding author (to get the data, click the link: https://www.jianguoyun.com/p/DQI3xkEQ0YfBChiW09EEIAA).
